# The Isolation of New Pore-Forming Toxins from the Sea Anemone *Actinia fragacea* Provides Insights into the Mechanisms of Actinoporin Evolution

**DOI:** 10.3390/toxins11070401

**Published:** 2019-07-10

**Authors:** Koldo Morante, Augusto Bellomio, Ana Rosa Viguera, Juan Manuel González-Mañas, Kouhei Tsumoto, Jose M. M. Caaveiro

**Affiliations:** 1 Department of Bioengineering, Graduate School of Engineering, The University of Tokyo, Bunkyo-ku, Tokyo 113-8656, Japan; 2 Department of Biochemistry and Molecular Biology, University of the Basque Country, P.O. Box 644, 48080 Bilbao, Spain; 3 Instituto Biofisika (CSIC, UPV/EHU), Parque Científico de la UPV/EHU, Barrio Sarriena s/n, 48940 Leioa (Bizkaia), Spain; 4 Instituto Superior de Investigaciones Biológicas (INSIBIO, CONICET-UNT) e Instituto de Química Biológica “Dr. Bernabé Bloj,” Facultad de Bioquímica, Química y Farmacia, Universidad Nacional de Tucumán, Chacabuco 461, T4000 San Miguel de Tucumán, Argentina; 5 Institute of Medical Science, The University of Tokyo, Minato-ku, Tokyo 108-8639, Japan; 6 Department of Global Healthcare, Graduate School of Pharmaceutical Sciences, Kyushu University, Fukuoka 812-8582, Japan

**Keywords:** actinoporins, protein variability, protein stability, protein structure, protein evolution

## Abstract

Random mutations and selective pressure drive protein adaptation to the changing demands of the environment. As a consequence, nature favors the evolution of protein diversity. A group of proteins subject to exceptional environmental stress and known for their widespread diversity are the pore-forming hemolytic proteins from sea anemones, known as actinoporins. In this study, we identified and isolated new isoforms of actinoporins from the sea anemone *Actinia fragacea* (fragaceatoxins). We characterized their hemolytic activity, examined their stability and structure, and performed a comparative analysis of their primary sequence. Sequence alignment reveals that most of the variability among actinoporins is associated with non-functional residues. The differences in the thermal behavior among fragaceatoxins suggest that these variability sites contribute to changes in protein stability. In addition, the protein–protein interaction region showed a very high degree of identity (92%) within fragaceatoxins, but only 25% among all actinoporins examined, suggesting some degree of specificity at the species level. Our findings support the mechanism of evolutionary adaptation in actinoporins and reflect common pathways conducive to protein variability.

## 1. Introduction

Protein toxins have diversified through evolution to acquire specialized functions such as predation, defense, and digestion [1,2,3,4]. This diversification is the result of different kinds of evolutionary adaptation. Some toxins have evolved under the influence of positive (diversifying) selection, which favors the rapid segregation of new phenotypes [5,6]. Others, such as cnidarian pore-forming toxins, are mostly influenced by negative (purifying) selection where proteins retain functionally important regions [7], leading to a wide distribution of highly similar protein species.

The multigene family of actinoporins are a group of cnidarian pore-forming toxins produced by sea anemones well known for their widespread polymorphism [8]. Strongly influenced by negative selection [3,7], actinoporins have conserved important functional sites to ensure the preservation of their mechanism of pore formation, a complex process involving many different steps [9,10]. Most notable is the surface-exposed lipid/carbohydrate-binding module involved in toxin binding to cell membranes [11]. Non-specific binding to membranes opens the possibility to target a wide range of species and may eliminate the need for the predator–prey chemical arms race [7,12]. Other regions that exhibit high conservation are the protein–protein binding surfaces that contribute to the oligomerization of membrane-bound actinoporin monomers [13]. Although previous studies have identified some variability in residues involved in protomer–protomer interaction [3], high-resolution crystallographic data indicate that the three-dimensional arrangement of the protein–protein interfaces is mostly conserved [11]. Regions contributing to actinoporin variability therefore are expected to be unrelated to function.

A previous study provided clues into the variability of non-functional residues using statistical inference analysis of selection rates of specific actinoporin gene clusters. Whereas actinoporins as a whole display high rates of negative selection, analysis of site-specific rates of evolution identified three sites under the influence of positive selection [3]. Interestingly, none of these sites (their exact location is not indicated in the referred study) corresponded to known functions.

Variability in sites with no specific function is not uncommon in nature. In fact, it is widely accepted that most mutations affect stability, whereas only a few are likely to affect function [14,15,16,17], most likely due, in part, to a higher mutational frequency in regions away from the functional binding surfaces. This may not be surprising, since only a minor fraction of the protein residues belongs to binding interfaces or catalytic sites, therefore reducing the chance for a random mutation to occur on that site. Most of the mutations that affect protein function are usually detrimental for the protein and are either purged away by natural selection or traded off with additional stabilizing mutations [18,19]. Moreover, protein evolution describes that stability is not necessarily associated with changes in protein function but is a general constraint [18,20,21], reducing furthermore the prevalence of the functional deterrent mutations with respect to those that are neutral or have meager effects in stability. As a result, protein variability is enhanced in residues with no specific function, most likely contributing to stability–structure relationships.

After the identification and isolation of a new actinoporin from the sea anemone *Actinia fragacea* [22], we studied the applicability of this concept to actinoporins and made a comparative analysis at the single-residue level. This analysis confirmed that the largest sequence variability came from residues/loci not involved in intermolecular interactions. In addition, the location of these residues in areas of partial solvent accessibility could explain the changes in protein stability observed among fragaceatoxin species and be valid for actinoporins in general.

## 2. Results

### 2.1. Purification of Fragaceatoxins

**Figure 1:**
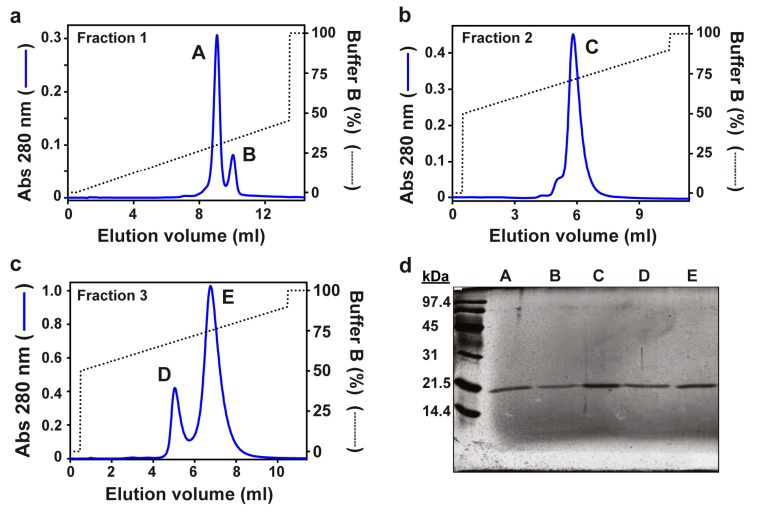
Purification of fragaceatoxins. Three hemolytic fractions (1, 2 and 3) resulted from the elution of the liquid exuded from *Actinia fragacea*, as reported earlier [22]. (**a**) Fraction 1 was loaded in a Mono S column, obtaining peaks A and B. The same treatment was carried out with fractions 2 and 3, resulting in the elution of (**b**) protein peak C and (**c**) protein peaks D and E, respectively. Blue traces correspond to the absorbance at 280 nm, and the dotted tracesindicate the percentage of buffer B. (**d**) SDS-PAGE of protein peaks A, B, C, D, and E (silver-stained).

Five actinoporins from *Actinia fragacea* were purified as described in Materials and Methods. Elution of *Actinia fragacea* venom through an SP Sepharose column rendered three hemolytic peaks [22]. These peaks were loaded in a Mono S 5/5 column, obtaining five elution peaks that we termed A, B, C, D, and E (Figure 1a–c). In particular, peak C corresponded to the well-characterized toxin fragaceatoxin C (FraC), which was used for comparative purposes in further analyses.

Samples of each protein peak were subjected to SDS-PAGE. Silver staining revealed single protein bands of approximately 20 kDa (Figure 1e), the molecular mass typical among actinoporins. Mass spectrometry confirmed the size of the new proteins and matched those in electrophoresis (Table 1 and Figure S1, Supplementary Materials). Single protein sequences were unveiled by Edman degradation, revealing unique protein sequences (Table 1). A BLAST search of the N-terminal sequences showed >60% identity with FraC, suggesting that these proteins are fragaceatoxin isoforms. The new proteins were accordingly named FraA, FraB, FraD, and FraE.

### 2.2. Hemolytic Activity

**Figure 2:**
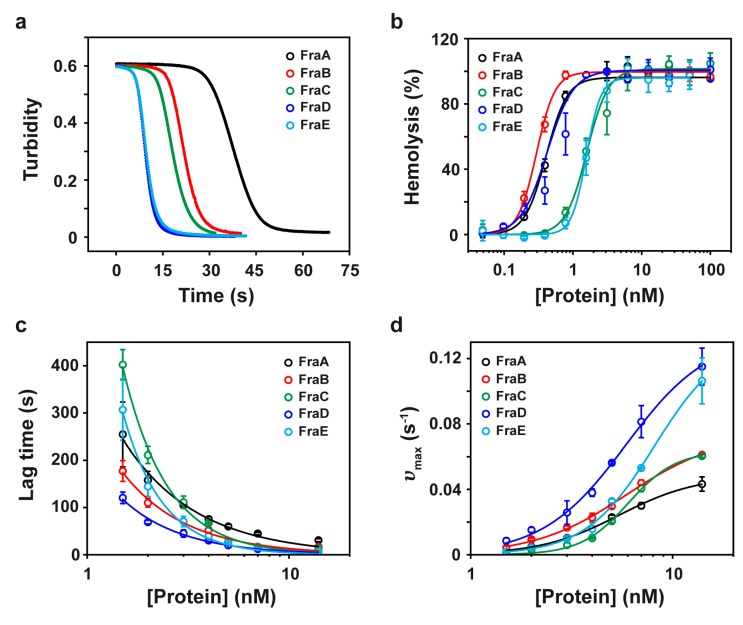
Hemolytic activity of fragaceatoxins. (**a**) Kinetic course of hemolysis by FraA, FraB, FraC, FraD, and FraE at 14 nM toxin. (**b**) Percentage of hemolysis of sheep red blood cells. Data were fitted to the Hill equation (solid traces). (**c**) Lag time as a function of protein concentration. Data points were fitted to a power law function [25]. (**d**) Representation of υ_max_ as a function of protein concentration. Data were fitted to the Hill equation (solid traces). In all panels, black, red, green, blue, and cyan traces correspond to FraA, FraB, FraC, FraD, and FraE, respectively.

Actinoporins exhibit a potent hemolytic behavior [23]. Hemolysis results from the formation of a functional pore in the red-cell plasma membranes that breaks the permeability barrier and gives rise to a colloid-osmotic shock. The formation of a functional oligomeric pore takes place before the onset of hemolysis during the so-called lag time. The steps leading to pore formation comprise (1) the binding of the toxins to the cell membrane, (2) toxin diffusion across the membrane plane, (3) protein oligomerization, and (4) translocation of the N-terminal α-helices through the lipid bilayer [10,24]. The parameter HC_50_ is defined as the concentration of protein producing 50% hemolysis. The most active toxins are characterized by short lag times and low HC_50_. Figure 2b and Table 1 show that FraA, FraB, and FraD are the most potent toxins within the fragaceatoxin species.

The hemolysis reaction can be followed by the changes in turbidity (absorbance at 700 nm) observed in a red cell suspension after addition of the toxins (Figure 2a). Reaction kinetics describe a characteristic sigmoidal curve governed by an initial lag time prior to lysis and a maximum velocity of hemolysis (υ_max_) (Figure 2c,d). Examination of these two parameters over a range of toxin concentrations revealed a decrease of the lag time concomitantly with an increase of υ_max_, indicating that these two parameters were somehow correlated. The rate of change in lag time as a function of protein concentration, however, clearly distinguished FraC and FraE (time constants ≈800) from the other fragaceatoxin isoforms (time constants ≈300) (Figure 2c).

By using a saturating protein concentration (14 nM), additional information of the lytic mechanism was obtained. At this concentration, there was enough protein for the monomers to rapidly bind to lipid molecules and oligomerize. Under these conditions, the rate-limiting step is the insertion of the N-terminal region in the hydrophobic core of the membrane. At saturating concentration, the most active toxins were FraD (lag time = 7 ± 0.8 s; υ_max_ = 115 ± 11 s^−1^) and FraE (lag time = 7 ± 1 s; υ_max_ = 106 ± 14 s^−1^), followed by FraC (lag time = 13 ± 0.4 s; υ_max_ = 60 ± 1 s^−1^), FraB (lag time = 17 ± 0.1 s; υ_max_ = 61 ± 1 s^−1^), and FraA (lag time = 31 ± 3 s; υ_max_ = 43 ± 4 s^−1^) (Figure 2c,d and Table 1).

### 2.3. Thermal Stability

**Figure 3:**
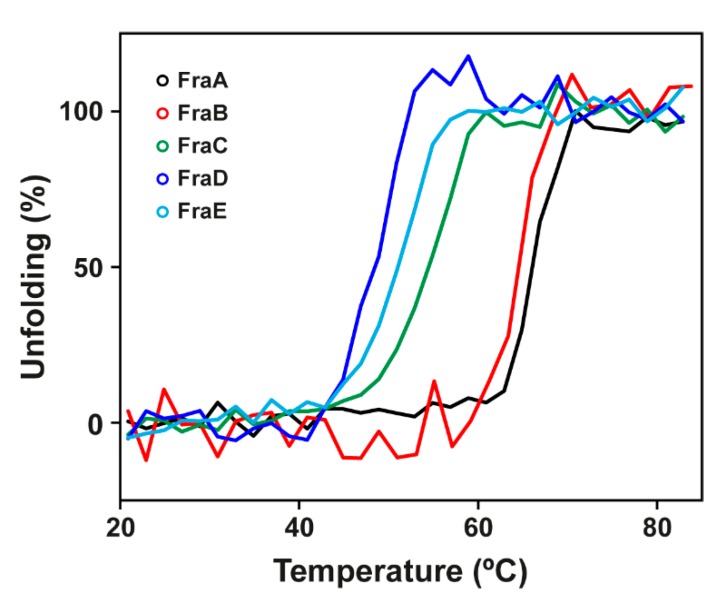
Thermal stability. Temperature-induced unfolding of fragaceatoxins monitored by circular dichroism at 210 nm. Black, red, green, blue, and cyan traces correspond to FraA, FraB, FraC, FraD, and FraE, respectively. Traces were smoothed using the group reduction function in OriginPro.

The thermal stability of the toxins was monitored by the change of ellipticity in the far-UV region (210 nm) with increasing temperature (Figure 3). The midpoint of the unfolding transition (*T_M_*) of the proteins was determined. The most stable isoforms were FraA and FraB as judged by their higher *T_M_* values (*T_M_*^FraA^ = 65 ± 1 °C, *T_M_*^FraB^ = 62 ± 2 °C), followed by FraC, FraD, and FraE (*T_M_*^FraC^ = 53 ± 3 °C, *T_M_*^FraD^ = 47 ± 1 °C, and *T_M_*^FraE^ = 51 ± 1 °C) (Table 1).

### 2.4. DNA Sequence of Fragaceatoxins

For comparison purposes, we tried to obtain the sequences of as many fragaceatoxins as possible. Following the protocol described in Materials and Methods, four different DNA sequences were elucidated. The N-terminus of two of the translated sequences overlapped with the Edman degradation products of FraB and FraE and, consequently, these sequences were assigned to them. A third sequence was assigned to FraC after sequence comparison and proteomic analysis, as described previously [22]. The fourth sequence identified did not perfectly align to any of the fragaceatoxins purified, and may well belong to yet another fragaceatoxin isoform present in the venom of *Actinia fragacea*. Amplicons corresponding to FraA or FraD were not found in the pool, possibly due to the hybridization of the polyT reverse primer with another adenine-rich stretch in the sequence. Comparison of the molecular masses calculated from the DNA sequence (FraB = 19672.3 Da; FraE = 19776.4 Da) with those obtained from mass spectrometry (FraB = 19672 ± 3; FraE = 19777.5 ± 3) validated the identification of the sequence of these isoforms (Table 1 and Figure S1, Supplementary Materials).

The cDNA sequences of FraB and FraE are shown in Figure S2, Supplementary Materials. The cDNA sequences, however, are partial because the first 35 and 32 nucleotides of FraB and FraE, respectively, corresponded to the primers used in the amplification (Table S1, Supplementary Materials). As a consequence, the first 12 amino acids of FraB and 11 amino acids of FraE are extracted from the Edman degradation products, whereas the rest of the protein sequence was obtained by direct translation from the DNA sequence. The DNA codifying sequences for FraB and FraE have been deposited in GenBank with accession numbers MK936900 and MK936901, respectively.

### 2.5. Structural Comparison of Actinoporins

**Figure 4:**
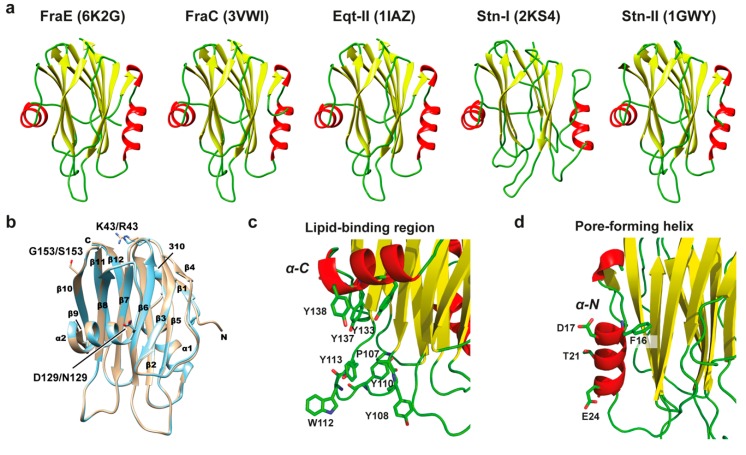
Three-dimensional structure of actinoporins. (**a**) Comparison of the crystal structure of FraE (6K2G, this work) with FraC (3VWI), Eqt-II (1IAZ), Stn-I (2KS4), and Stn-II (1GWY). Actinoporins form a characteristic β-sandwich flanked by two α-helices. Random coils are depicted in green, α-helices in red, and β-sheets in yellow. (**b**) Secondary structure elements in the structures of FraC (PDB entry code 3VWI, gold) and FraE (PDB entry code 6K2G, cyan), which were superimposed for the figure. The secondary structure elements β-sheet (β), α-helix (α), and 3_10_ helix (310) are indicated. The side chains of the variable residues Lys/Arg43, Asp/Asn129, and Gly/Ser153 are also shown. Letters C and N refer to the C- and N-terminus, respectively. (**c**) Close-up view of the lipid-binding region in FraE comprising a large number of aromatic residues along the C-terminal α-helix and a protruding hydrophobic loop. (**d**) N-terminal region in FraE depicting the N-terminal α-helix involved in membrane insertion during pore formation. The figure was made with CHIMERA [27].

To investigate the structural differences of FraB and FraE with other actinoporins, their primary sequences were aligned against the non-redundant protein database with BLASTP [26]. The search delivered sixteen proteins with >55% identity, all of which belonged to the actinoporin family. The high sequence identity anticipated a common three-dimensional structure. Indeed, the crystal structure of FraE (solved for the first time in this work at 2.2 Å) displayed a three-dimensional fold similar to that of other members of the actinoporin family such as FraC (3VWI; RMSD = 0.28 ± 0.03 Å), equinatoxin II (1IAZ; RMSD = 0.49 ± 0.13 Å), and sticholysin II (1GWY; RMSD = 0.59 ± 0.04 Å) (Figure 4). The structure is composed of a β-sandwich core flanked by an N-terminal and a C-terminal α-helix. Relevant functional areas such as the lipid-binding region (contoured by a hydrophobic loop and the C-terminal α-helix) and the pore-forming N-terminal α-helix were conserved. The preservation of actinoporin structure reflects the evolution under the constraints of negative selection [7].

**Figure 5:**
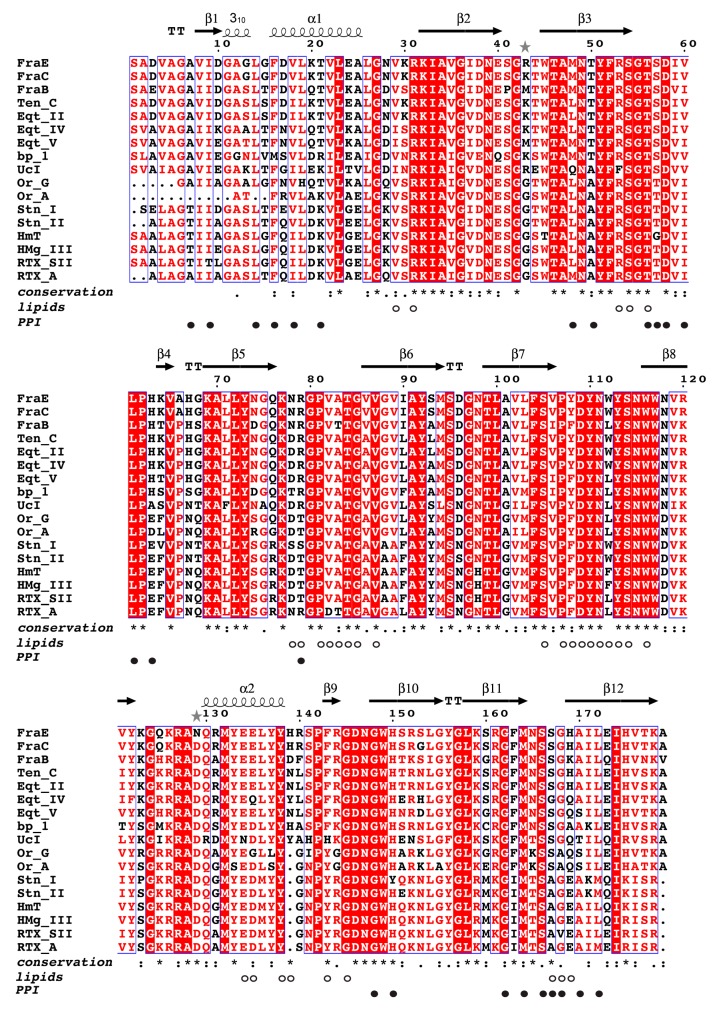
Multiple sequence alignment of actinoporins. Alignment of seventeen actinoporin sequences with >60% identity: FraB (MK936900), FraE (MK936901), and FraC (B9W5G6) from *Actinia fragacea*; tenebrosin-C (Ten-C, P61915) from *A. tenebrosa*; equinatoxin II (Eqt-II, P61914), equinatoxin IV (Eqt-IV, Q9Y1U9), and equinatoxin V (Eqt-V, Q93109) from *A. equina*; bandaporin (bp-1, C5NSL2) from *Anthopleaura asiatica*; cytolysin UcI (P0CG44) from *Urticina piscivora*; cytolysin Or-G (Q5I2B1) and cytolysin Or-A (Q5B4I8) from *Oulactis orientalis*; sticholysin I (Stn-I, P81662) and sticholysin II (Stn-II, P07845) from *Stichodactyla helianthus*; cytolysin HmT (P0DMX2) and magnificalysin III (HMg-III, Q9U6X1) from *Heteractis magnifica*; and cytolysin RTX-SII (P0C1F8) and cytolysin RTX-A (P58691) from *Heteractis crispa*. The alignment was built with webPRANK [28] and colored in ESPript 3.0 [29] by conservation: red background, red letters, and black letters denote the degree of conservation of single residues in descending order. The degree of conservation at each position was determined with Clustal Omega [30]: identical (*), strongly (:), and weakly conserved (.). Non-conserved positions are represented by an empty space. White and black circles indicate residues involved in lipid–protein (*lipids*) and protein–protein interactions (*PPI*) [10]. The secondary structure elements β-sheet (β), α-helix (α), and 3_10_ helix (310) are depicted above the sequences and correspond to those shown in Figure 4b. Turns are labelled with the letter T, and loops are not labeled. Gray stars indicate different residues between FraC and FraE.

The sequence alignment of 17 actinoporins evidenced the high similarity among them (Figure 5). When considering only the differences among fragaceatoxins, it is observed that FraE shared a high identity (98%) with FraC, explaining their similar activity above (Figure 2b). Although FraB displayed greater differences (37 different residues), the degree of identity with either FraC or FraE was still significantly high (≈80%). Most of these 37 residues were not involved in specific binding functions. Only 5 of these 37 different residues (14%) were involved in protein–protein (Val60 and His169 of FraC) and protein–lipid interactions (Ala83, Tyr108, and Trp112 of FraC). The other 32 residues accounted for 86% of the differences but did not exhibit interacting partners. Given the noted differences in sequence, activity, and stability between FraB and FraE, we considered these as clear representatives of fragaceatoxin variability and selected them for further analysis.

When all known actinoporins were compared, the number of non-identical residues increased for both the non-interacting residues (from 32 to 84 non-identical residues) and for the residues involved in lipid–protein and protein–protein interactions (from 5 to 30 non-identical residues). When we look at the degree of identity within each category, it is observed that residues involved in lipid–protein interactions are the most conserved (56%). In contrast, only 25% of the residues involved in protein–protein interactions were identical, indicating that protein–protein interaction surfaces are evolutionarily more variable than lipid–protein surfaces. Functional differences between actinoporins may thus rely greatly on both non-interacting residues and in residues belonging to the protein–protein interface.

### 2.6. Distribution of Actinoporin Variability

**Figure 6:**
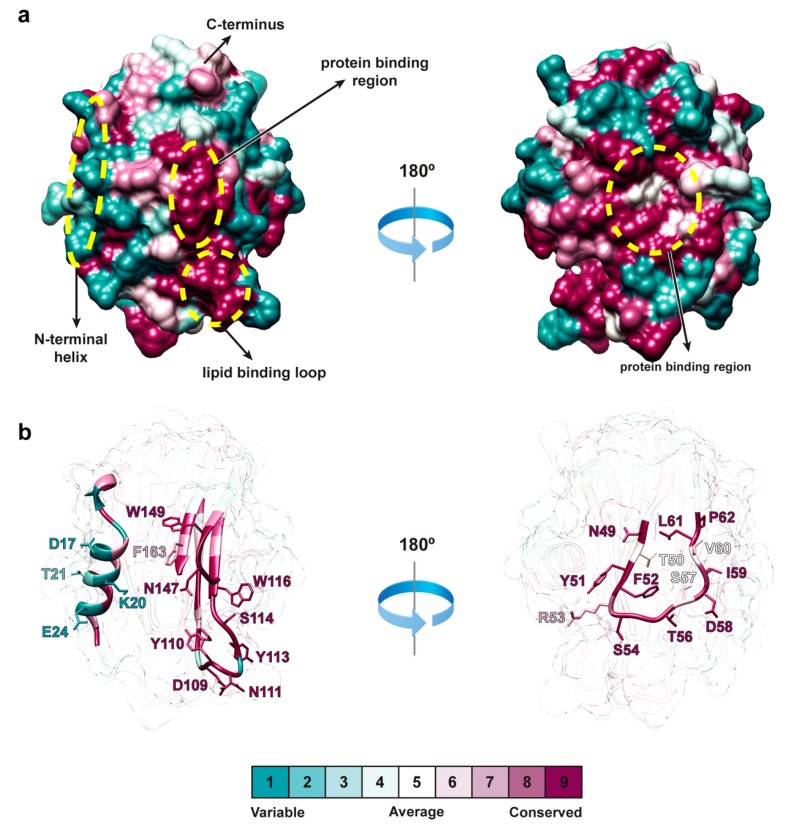
Evolutionary variability of actinoporins. (**a**) Surface area representation of FraE (PDB code 6K2G, this work) color-coded according to the evolutionary variability of actinoporins (see color legend). The N-terminal helix, the protein-binding region and the lipid-binding region are circled in yellow. These three regions are shown in ribbon representation in (**b**). Mapping the evolutionary variability of residues on the crystal structure was made in the Consurf server [33].

The largest number of identical or highly conserved regions in proteins are generally found in binding interfaces or protein cores where the accessible surface area (ASA) of residue side chains is low (Figure 6). Conversely, non-conserved residues are found in regions with high ASA (away from binding interfaces and protein cores). To study whether actinoporins follow this distribution, residues were classified according to their degree of conservation and ASA. The most abundant populations corresponded to identical residues with low ASA (35 residues) and to non-conserved residues with high ASA (26 residues) (Table S3, Supplementary Materials), where the former reflected the negative selection rates common to actinoporins. Another group exhibiting a large contribution to variability corresponded to a group of 22 non-conserved residues with partially exposed side chains. Comparably, non-conserved residues in the group of fragaceatoxins are distributed in a similar ratio of 12 highly and 9 partially exposed residues.

The analysis for the 127 non-interacting residues revealed that the conservation/ASA distribution of the non-interacting residues closely resembles that of the overall residues—the most abundant group corresponded to identical residues with low ASA (24 residues) and non-conserved with high ASA (22 residues), whereas a relatively large variability came from the group of partially exposed and non-conserved residues (13 residues) (Table S4, Supplementary Materials). Similarly, the number of non-interacting residues with high ASA with respect to those with partial ASA in the family of fragaceatoxins (12 to 7) suggested that these toxins are good representatives of the variability seen in actinoporins.

Among the interacting residues, a high number of identical buried residues (11 residues) and partially buried side chains (9 residues) were observed (Table S5, Supplementary Materials). Of those, seven residues belong to protein–protein interacting surface (Table S6, Supplementary Materials), whereas only two belong to the lipid-interacting residues (Table S7, Supplementary Materials). In stark contrast, fragaceatoxins show practically no variability among the interacting residues, especially those involved in protein–protein interactions. This could point to the specificity of protein–protein interactions between members of the same species as has been observed before in sticholysin heteropores [31,32], a hypothesis that must be verified after a more complete examination of actinoporins using a greater number of toxin isoforms.

### 2.7. Evolutionary Divergence of Fragaceatoxins

**Figure 7:**
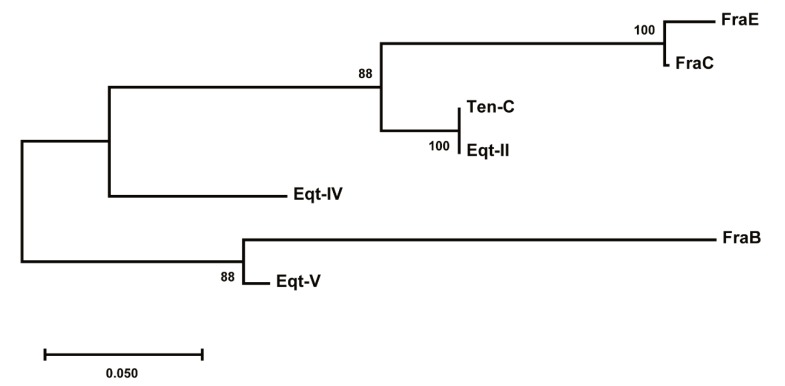
Evolutionary divergence tree of actinoporins from the genus *Actinia*. One cluster groups FraE, FraC, tenebrosin C (Ten-C), equinatoxin II (Eqt-II), and equinatoxin IV (Eqt-IV) and a second cluster groups FraB and equinatoxin V (Eqt-V). The length of the branches (number of substitutions per site expressed as parts per unit) determines the evolutionary distance from the common ancestor until speciation or until present. Confidence levels to assess the degree of relatedness of the proteins in a clade (bootstrap values) are shown next to the branches. The tree was built with MEGA X [34].

Phylogenetic analysis of the actinoporins from the genus *Actinia* shows the evolutionary divergence of this group of proteins (Figure 7). Apparently, a gene duplication event prior to speciation within the family gave rise to two different clusters. The first cluster groups the actinoporins with a higher degree of sequence identity (>78%), where the differences might have arisen during the speciation process without many changes. The other cluster contains FraB and equinatoxin V (Eqt-V), two actinoporins that also evolved through speciation but derive from one copy of the gene subjected to greater evolutionary change and, thus, sharing less identical residues with their respective isoforms. As a result, in some cases there is more degree of identity among different species of the same genus than among the different isoforms within one single species.

## 3. Discussion

The majority of randomly acquired mutations in proteins are neutral or weakly destabilizing [17] and generally occur in solvent-exposed regions [35]. These observations are consistent with neutral theories of molecular evolution and explain the general trend in the genetic variability of organisms [36,37,38,39]. In agreement with these trends, we have observed that variability in actinoporins occurs more frequently in non-functional regions away from binding interfaces and protein cores.

Random mutations in non-functional and solvent-exposed regions commonly accumulate through evolution, producing small changes in protein stability. These changes accumulate and drive the protein along a stability path delimited by deleterious hyper- and hypo-stability boundaries, above which the protein loses function [18,21]. Whether the protein crosses the boundaries depends on both the current stability level of the protein and the magnitude of stability change brought by a new mutation which, in turn, depends on the type of mutation and the region of the protein affected. For this reason, most of the identical or conserved residues are found in buried areas (protein cores and interfaces) and most of the non-conserved residues are found in solvent-exposed surfaces not associated with binding sites.

Our study unveiled that actinoporins reflect this distribution and, whereas protein–protein interactions have some contribution to the variability of actinoporin function, the more variable non-interacting sites may tweak protein stability (Figure 3) and potentially influence protein activity. This conclusion is based on the fact that one-third of non-conserved non-interacting residues in actinoporins (13 out of 40, Table S4, Supplementary Materials) are at least partially buried, thus having a higher chance to influence protein stability than the more abundant but highly exposed non-conserved residues. Additionally, the fact that this ratio closely resembles that found among the total number of residues (22 out of 58 or 38%, Table S3, Supplementary Materials) indicates that the conservation/ASA distribution of non-interacting residues is representative of actinoporins. Interestingly, fragaceatoxins follow a similar distribution of non-interacting (7 out of 21 or 33%, Table S4, Supplementary Materials) and total residues (9 out of 23 or 39%, Table S3, Supplementary Materials), providing a suitable model for actinoporin variability. The fact that the variability displayed by fragaceatoxins (86% attributed to non-interacting residues) may lie behind the differences in stability (Figure 3) suggests that this may also happen in actinoporins in general as the percentage of non-interacting residues that contribute to actinoporin variability is comparable (74%). This observation suggests that non-interacting residues accumulate to give an overall effect in the stability, in agreement with previous proposals [17].

Determining the specific effects of these mutations on protein activity, however, is a difficult task, especially considering that, although fewer in number, mutations in interacting residues have a direct impact on protein’s function and these effects may eclipse those away from the functional binding sites. The effects of the overall substitutions are best evidenced in the lag-time variations between the toxin isoforms, given that the sequence of events leading to pore formation takes place during this interval, and may more faithfully resemble the overall protein activity. Differences in the exponential dependencies of the lag time on the protein concentration distinguish the more cooperative FraC and FraE from the other fragaceatoxin isoforms (Figure 2c). In particular, these two proteins are predicted to have regions of larger, local intrinsic disorder (Figure S3, Supplementary Materials [40]), suggesting an underlying local flexibility that may enhance binding cooperativity. The overall hemolytic activities, however, are lower, implying that protein flexibility may obstruct other steps during the pore-forming process.

The study of pore formation can be further simplified at saturating protein concentrations where the detachment and insertion of the N-terminal helix in the membrane becomes rate-limiting. Attributing differences in activity to the N-terminal helix is evident, given its high sequence variability (Figure 5 and Figure 6). Careful examination of the hemolysis curves at saturating protein concentrations of fragaceatoxins (14 nM), nevertheless, shows meager differences between FraB and FraC, despite having seven different residues in the N-terminal helix (residues 1–29). Moreover, FraC and FraE share an identical N-terminal helix, although the hemolysis produced by the latter is considerably faster (Figure 2a). These experiments reveal that the differences in the N-terminal helix between fragaceatoxins are not responsible for the kinetic differences observed. Other effects such as protein stability or flexibility may be implicated.

Given the high sequence similarity between FraC and FraE, other possibilities such as long-range interactions may help to explain kinetic differences, such as those described for Stn-II [41,42]. In this study, surface-exposed mutation R29Q in Stn-II was suggested to be responsible for long-range electrostatic interactions on membrane targeting and protein dynamic flexibility. Similar mechanisms may apply to the arginine/aspartate difference at position 129 of FraC versus FraE. The resulting loss of negative potential could favor the attraction of the protein to the negatively charged phospholipids in the membrane. In another study, a K159E substitution on a D10R FraC DNA translocation-optimized mutant recovered the hemolytic activity of the toxin [43], suggesting the influence of long-range interactions between these distant residues. Interestingly, the specific substitution was discovered by random mutagenesis, which serves as an example of how random mutations can successfully compensate deleterious mutations in actinoporins, reflecting natural evolution.

## 4. Conclusions

Overall, our results are in line with the mechanisms of evolutionary adaptation of actinoporins where functional and structural motifs are strongly conserved. Herein, we have revealed that most of the residues contributing to actinoporin variability correspond to sites with no specific function, possibly related to protein stability, which may in turn influence protein function. In consequence, our results potentiate the view where actinoporin evolution is limited by the stabilizing/destabilizing effects of single residue substitutions, reflecting a mutagenic pathway common in the evolution of proteins that forms the basis of actinoporin diversity.

## 5. Materials and Methods

### 5.1. Purification of Fragaceatoxins

Fragaceatoxins were obtained from sea anemones as described previously [22]. Briefly, the venom expelled from the animal’s body was subject to cation-exchange chromatography through an SP Sepharose Fast Flow column (GE Healthcare, Piscataway, NJ, USA). Three hemolytic peaks were collected in three separate fractions (1, 2, and 3) and independently loaded on a Mono S HR 5/5 column (GE Healthcare) equilibrated in 50 mM sodium acetate buffer pH 5 (buffer A). Mono S has a higher resolution to separate proteins and was used to isolate fragaceatoxin isoforms. Proteins in fraction 1 were eluted with 50 mM sodium acetate buffer, 1 M NaCl pH 5 (buffer B) using a linear gradient from 0 to 45% buffer B, and produced two peaks termed A and B. Fraction 2 was eluted with a gradient from 50% to 90% buffer B and produced one peak termed C, corresponding to FraC. The same salt gradient was used to elute fraction 3, obtaining two additional peaks called D and E. Protein purity was assessed by SDS-PAGE and the concentration determined by the Bradford assay [44].

### 5.2. N-Terminal Sequencing

Fragaceatoxins were run on an SDS-PAGE and then transferred to an Immobilon-P polyvinylidene fluoride membrane (Merck Millipore, Darmstadt, Germany) and stained with 0.1% Coomassie in water/methanol/acetic acid (50:40:10) to localize the protein bands. Membranes were air dried, bands were excised, and their 20 N-terminal residues were sequenced by Edman degradation [45].

### 5.3. Hemolytic Activity

Sheep red blood cells (Pronadisa, Madrid, Spain) were washed by centrifugation at 1200× *g* for 10 min with 150 mM NaCl, 5 mM sodium phosphate pH 8 buffer (hemolysis buffer), and resuspended in buffer to a turbidity value of 0.6 when measured at 700 nm with a spectrophotometer. Hemolysis was measured by monitoring changes in turbidity after addition of the toxin. Experiments were carried out at 25 °C with constant stirring. Kinetics of hemolysis displayed a characteristic sigmoidal curve where the maximum slope was used as a measure of the maximum velocity of hemolysis (υ_max_). The time taken from toxin addition to the onset of hemolysis was used as a measure of the lag time. To judge the change of lag time as a function of toxin concentration, time constants were extracted using a power law equation [25]:

lag time = τ · c^−x^, (1)

where τ is the time constant, *c* is the protein concentration, and *x* is the order of the reaction.

In the experiments performed to measure the hemolytic potency of the toxins (concentration dependence), a population of red blood cells was prepared such that 37.5 μL of erythrocytes gave a reading of A_412_ = 0.6 in distilled water. Two-fold serial dilutions of the toxins in hemolysis buffer were made in microtiter plates and then an equal volume of red blood cells was added to start the reaction. Control rows with no toxin and distilled water were made to determine the A_412_ at 0% (A_final_) and 100% (A_max_) hemolysis, respectively. The mixture was incubated for 90 min at 25 °C spinning at 300 rpm on a Thermomixer (Eppendorf, Hamburg, Germany). After this time, the plates were centrifuged at 2000× *g* for 10 min to pellet not lysed cells and the supernatant was recovered to measure A_412_. The extent of hemolysis for each concentration was calculated according to the following equation:

Hemolysis (%) = [(A_412_ − A_fin_)/(A_max_ − A_fin_)] × 100. (2)

The percentage of hemolysis was plotted as a function of the protein concentration. The toxin concentration that produced 50% hemolysis (HC_50_) was obtained by fitting the Hill equation [46] to the calculated data points.

### 5.4. Protein Stability

Protein unfolding was monitored by circular dichroism in a J-810 spectropolarimeter (JASCO, Tokyo, Japan) equipped with a Jasco PTC-423 temperature controller. The sample was diluted to 1–2 μM in 20 mM Tris-HCl, pH 8.0 and denaturation curves recorded at a wavelength of 210 nm from 10 to 90 °C at a heating rate of 1 °C/min. The bandwidth used was 2 nm. Melting temperatures (*T_M_*) were calculated as the maxima of the first derivatives of the percentage of change of ellipticity at 210 nm versus temperature curves. For a clear depiction of the kinetic traces, data points were smoothed by the group reduction function implemented in OriginPro software. This function calculates new *x* and *y* values from the average of ten *x*-axis and ten *y*-axis points, respectively.

### 5.5. Cloning and DNA Sequence Determination

RNA was purified from *A. fragacea* by the guanidine thiocyanate–phenol–chloroform extraction method [47] and was then used as template for RT-PCR using the QIAGEN OneStep RT-PCR Kit (Qiagen, Valencia, CA, USA). The oligonucleotide primers were those previously used for the amplification of fragaceatoxin sequences [22] and are shown in Table S1, Supplementary Materials. The cDNA amplicons of ≈750 bp were cloned into a pGEM-T Easy Vector (Promega, Madison, WI, USA) for PCR products using standard methodologies [48]. Transformation of the *Escherichia coli* XL1-Blue strain with the ligation mixture allowed the white/blue selection of bacterial colonies grown in the presence of X-Gal. Restriction analysis and DNA sequencing of the selected plasmids further confirmed the existence of the inserts. The DNA codifying sequences for FraB and FraE have been deposited in GenBank under accession numbers MK936900 and MK936901, respectively.

### 5.6. Cloning, Expression, and Purification of FraE

Cloning of FraE into the pBAT4 expression vector was made with the In-Fusion HD Cloning Kit (Takara Bio Company, Shiga, Japan). Briefly, two independent PCRs were made to amplify the FraE gene and the destination vector in linearized form. The destination vector used was the pBAT4-based expression vector of FraC produced previously [22]. The purified PCR products were then mixed and incubated with In-Fusion enzyme premix for ligation. The oligonucleotide primers for the FraE gene and the destination vector were designed with 15 bp extensions homologous to vector ends to permit reliable hybridization of insert and vector during ligation (Table S1, Supplementary Materials). To enable the expression of FraE in *E. coli*, the forward primer used to amplify FraE was engineered to substitute the first amino acid of FraE (serine) to methionine. The DNA sequence used for FraE was codon-optimized to improve expression of the protein in *E. coli* necessary for crystallography experiments.

Expression and purification of FraE were carried out essentially as described in [22] with some modifications. *E. coli* BL21(DE3) cells were chemically transformed with the FraE expression vector and grown to OD_600_ = 0.5 at 37 °C. Expression was induced with 0.5 mM isopropyl β-D-1-thiogalactopyranoside for 20 h at 20 °C and cells were harvested by centrifugation at 8000× *g* for 10 min at 4 °C. Pelleted cells were resuspended in cold Tris 50 mM, pH 7.4 (buffer A), and lysed with a probe sonicator. The bacterial lysate was centrifuged at 40,000× *g* for 30 min at 4 °C, treated with 0.01 mg/mL DNase (30 min at 25 °C), and filtered sequentially through a 0.45 μm pore-size Sterivex-HV filter followed by a Millex-GP 0.22 μm filter unit (Merck Millipore). The filtrate was loaded into a Resource S cation-exchange column (GE Healthcare) equilibrated in buffer A and washed with 50 mL of 10% Tris 50 mM, 1 M NaCl pH 7.4. Toxin elution was achieved by a linear gradient of Tris 50 mM, 1 M NaCl pH 7.4 (buffer B) applied over 60 mL. Fractions corresponding to the largest peak were concentrated and applied to a HiLoad 16/60 Superdex 75 prep grade column (GE Healthcare) equilibrated in Tris 50 mM, 200 mM NaCl pH 7.4. FraE was eluted after one column volume.

### 5.7. Crystallization of FraE

To obtain crystals of FraE suitable for X-ray diffraction, the protein was dialyzed against 10 mM Tris-HCl pH 8. Crystal screening was made using a PEG/Ion reagent kit (Hampton Research, Aliso Viejo, CA, USA) by systematically mixing 1 μL protein at 9 mg/mL with 1 μL crystal solution using an Oryx8 robot (Douglas Instruments, Hungerford, UK). Crystals of FraE grew on 0.2 M sodium formate, 20% *w*/*v* PEG 3350 pH 7.2. Crystals were transferred to a solution of mother liquor supplemented with 20% (*v*/*v*) glycerol and fast frozen in liquid nitrogen. Diffraction data was collected at Beamline BL5A of the Photon Factory (Tsukuba, Japan) under cryogenic conditions (100 K). Diffraction images were processed to 2.2 Å resolution with the program MOSFLM and merged and scaled with the program SCALA [49] of the CCP4 suite [50]. The crystal structure of FraE was solved by molecular replacement using the coordinates of FraC (PDB code 3VWI) with the program PHASER [51]. The model was refined with REFMAC5 [52] and COOT [53]. Validation was carried out with PROCHECK [54]. Data collection and refinement statistics are given in Table S2, Supplementary Materials.

### 5.8. Mass Spectrometry

For the determination of the molecular masses of fragaceatoxin isoforms, each protein (≈20 μg) was desalted and the buffer was exchanged to 50% acetonitrile, 0.2% formic acid using a Micro Bio-Spin chromatography column (Bio-Rad, ). The protein was directly injected into a Q-Tof Micro mass spectrometer (Waters, Milford, MA, USA) and spectra were manually acquired in the *m*/*z* range of 700–2500. The mass of the protein was determined by MaxEnt1 software (Waters) using the default deconvolution parameters provided by the program. and the software was set to iterate to convergence. Modifications such as sodium and potassium adducts or methionine oxidation were also taken into account for the calculation of the theoretical masses.

### 5.9. Primary Structure Analyses

Toxins FraB and FraE were used as query sequences in the protein–protein BLAST program [55] to retrieve similar sequences from the non-redundant protein database. Sequence alignment was performed with a phylogeny-aware gap placement algorithm [56] in the webPRANK server [28] and colored with ESPript 3.0 [29]. The degree of conservation was calculated with Clustal Omega [30]. To build a phylogenetic tree of the actinoporins of the genus *Actinia*, aligned sequences of the genus *Actinia* were first analyzed with ProTest [57] to give a best fit to the Le-Gascuel (LG) model for protein evolution. Tree reconstruction was then made in MEGA X [34] using the maximum likelihood method and LG model [58,59] using 100 bootstrap replications and a Gamma site rate distribution with four discrete categories.

Mapping of the evolutionary variability of amino acids onto the crystal structure of FraE was performed in the Consurf webserver [33] using the maximum likelihood method and LG evolutionary model. GETAREA [60] was used to calculate the ASA ratio between the ASA of the residue side chain and the ASA of the residue in random coil conformation. For the ASA calculation, we used the atom coordinates of the crystal structure of FraC (4TSY).

## Figures and Tables

**Table 1:** Characteristics of fragaceatoxins.

Name	N-Terminal Sequence	Mass ^a^	HC_50_ (nM) ^b^	Lag (s) ^c^	υ_max_ (s^−1^) ^c^	*T_M_* (°C)
FraA	SAEVAGAVIEGAKLTFNVLQ	19,728 ± 3	0.4 ± 0.02	31 ± 3	43 ± 4	65 ± 1
FraB	SAEVAGAIIDGASLTFDVLQ	19,672 ± 3	0.3 ± 0.02	17 ± 0.1	61 ± 1	62 ± 2
FraC ^d^	SADVAGAVIDGAGLG	19,720 ± 3	1.6 ± 0.3	13 ± 0.4	60 ± 1	53 ± 3
FraD	SVAVAGAVIKGAALTFNILQ	19,721 ± 3	0.4 ± 0.3	7 ± 0.8	115 ± 11	47 ± 1
FraE	SADVAGAVIDGAGLGFDVLK	19,778 ± 3	1.6 ± 0.2	7 ± 1	106 ± 14	51 ± 1

^a^ Determined by mass spectrometry. ^b^ Values ± SE. ^c^ The values of lag time and υ_max_ were determined from three independent measurements employing 14 nM toxin (Figure 2b) ± SD. ^d^ The N-terminal sequence of FraC was previously reported [22].

## Supplementary Materials

The following are available online at https://www.mdpi.com/2072-6651/11/7/401/s1; Figure S1: Deconvoluted mass spectrum of fragaceatoxins on the true mass scale, Figure S2: Partial cDNA and amino acid sequence for fragaceatoxin B and E, Figure S3: Structure of actinoporins, Table S1: Primer sequences used in the amplification of fragaceatoxin sequences, Table S2: Data collection and refinement statistics, Table S3: Number of residues classified according to conservation and accessible surface area (ASA), Table S4: Number of non-interacting residues classified according to conservation and accessible surface area (ASA), Table S5: Number of interacting residues classified according to conservation and accessible surface area (ASA), Table S6: Number of protein-binding residues classified according to conservation and accessible surface area (ASA), Table S7: Number of lipid-binding residues classified according to conservation and accessible surface area (ASA).
