# Supplementary material for: The Isolation of New Pore-Forming Toxins from the Sea Anemone Actinia fragacea Provides Insights into the Mechanisms of Actinoporin Evolution
Source: Toxins (Basel). 2019 Jul 10;11(7):401. doi: 10.3390/toxins11070401 (PMC6669745; doi:10.3390/toxins11070401)
Supplement: Supplementary file 1 [file toxins-11-00401-s001.pdf]

Supplementary Materials: The Isolation of New Pore-Forming Toxins from the Sea Anemone Actinia fragacea Provides Insights into the Mechanisms of Actinoporin Evolution

Koldo Morante, Augusto Bellomio, Ana Rosa Viguera Juan Manuel González-Mañas, Kouhei Tsumoto and Jose M. M. Caaveiro

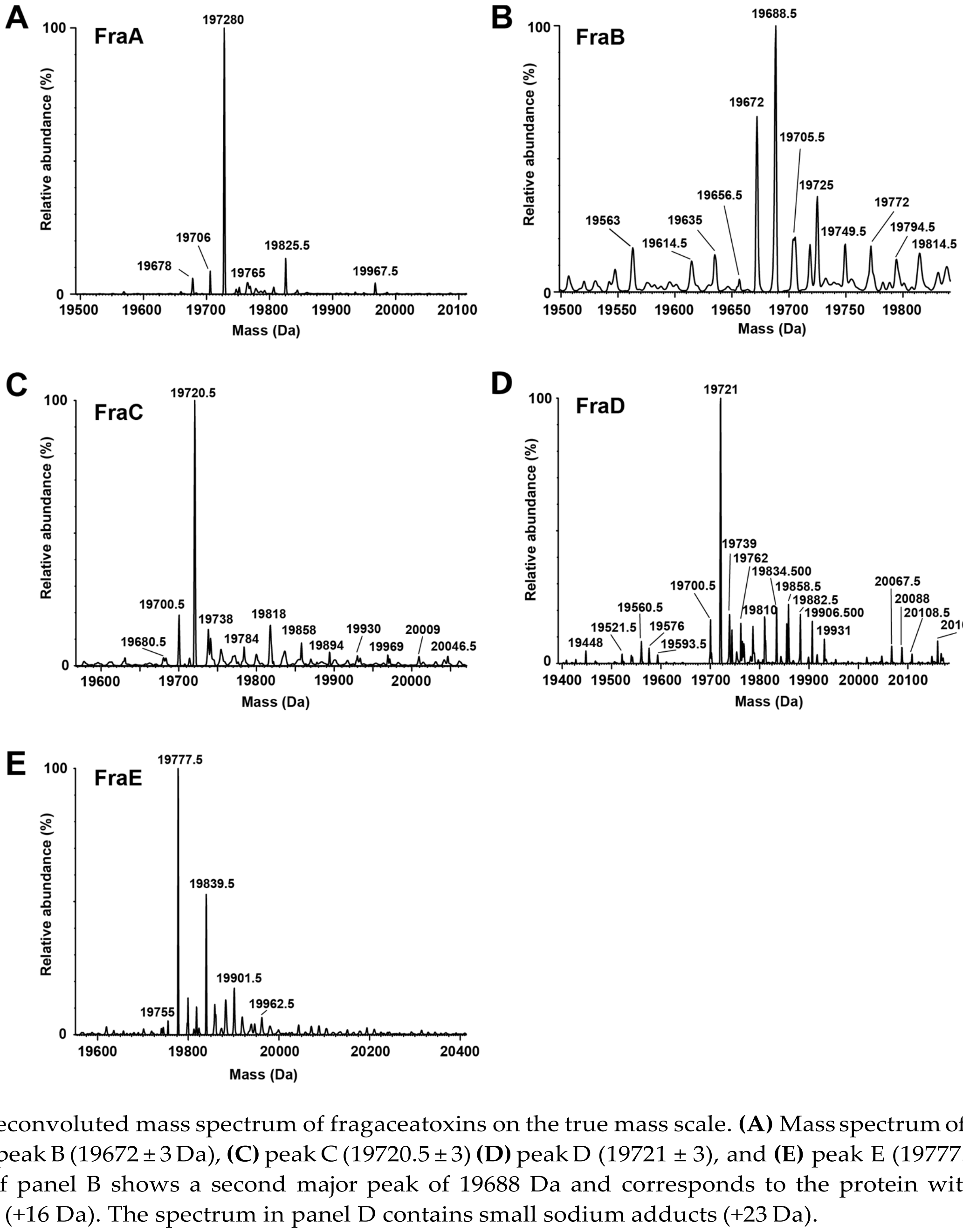

Figure 1. Deconvoluted mass spectrum of fragaceatoxins on the true mass scale. **(A)** Mass spectrum of peak A (19728 ± 3 Da), **(B)** peak B (19672 ± 3 Da), **(C)** peak C (19720.5 ± 3) **(D)** peak D (19721 ± 3), and **(E)** peak E (19777.5 ± 3 Da). The spectrum of panel B shows a second major peak of 19688 Da and corresponds to the protein with an oxidized methionine (+16 Da). The spectrum in panel D contains small sodium adducts (+23 Da).

A

```
                            S  A  E  V  A  G  A  I  I  D  G  A       12
     S  L  T  F  D  V  L  Q  T  V  L  K  A  L  G  D  V  S  R  K      32
1    AAGTCTGACCTTCGACGTCCTGCAGACCGTGCTCAAAGCACTCGGTGATGTCAGTAGAAAG
     I  A  V  G  I  D  N  E  P  G  M  T  W  T  A  M  N  T  Y  F      52
62   ATTGCCGTCGGTATCGACAACGAGCCGGGCATGACGTGGACCGCAATGAACACATACTTC
     R  S  G  T  S  D  V  I  L  P  H  T  V  P  H  S  K  A  L  L      72
122  CGTTCTGGTACCTCTGATGTCATCCTTCCCCATACAGTTCCACATAGTAAGGCACTGCTC
     Y  D  G  Q  K  N  R  G  P  V  T  T  G  V  V  G  V  I  A  Y      92
182  TACGACGGTCAGAAAAATCGTGGTCCAGTTACGACTGGCGTGGTTGGAGTAATTGCTTAT A  M
        S  D  G  N  T  L  A  V  L  F  S  I  P  F  D  Y  N  L         112
242  GCCATGAGTGATGGAAACACCCTGGCCGTTTTATTCAGCATTCCCTTTGACTATAACCTG
     Y  S  N  W  W  N  V  K  V  Y  K  G  H  R  R  A  D  Q  A  M      132
302  TACAGCAACTGGTGGAATGTCAAGGTCTATAAAGGACATAGACGAGCAGACCAGGCGATG Y  E
        E  L  Y  Y  D  F  S  P  F  R  G  D  N  G  W  H  T  K         152
362  TACGAGGAACTCTACTACGATTTCTCTCCATTTCGAGGGGACAATGGCTGGCACACCAAG
     S  I  G  Y  G  L  K  G  R  G  F  M  N  S  S  G  K  A  I  L      172
422  AGCATTGGATATGGGTTGAAAGGCCGTGGATTCATGAACAGCTCTGGAAAAGCCATACTG
     Q  I  H  V  N  K  V  *                                          179
482  CAAATTCACGTGAACAAAGTTTGAGGTCTTGTTGAAAACAAATCAGTTGAAATGCTGCCT
542  CGAGAATACTGATGTAAAACTAGCAATAAATTATAATTTTACCCTGTAAGAACAAGAAAA
602  CTAGATCTTCCCGTAACATAAAGACGAATAAAACGAAGCACCCGAAAAAAAAAAAAAAAA
662  AAAAAATAGGGATCCAATCAG
              BamHI
```

B

```
                               S  A  D  V  A  G  A  V  I  D  G       11
     A  G  L  G  F  D  V  L  K  T  V  L  E  A  L  G  N  V  K  R      31
1      TGCAGGTCTGGGCTTCGACGTCCTGAAAACCGTGCTCGAAGCACTCGGTAATGTCAAACGA
     K  I  A  V  G  I  D  N  E  S  G  R  T  W  T  A  M  N  T  Y      51
62     AAGATTGCCGTCGGTATCGACAACGAGTCGGGCAGGACGTGGACCGCAATGAACACATAC
     F  R  S  G  T  S  D  I  V  L  P  H  K  V  A  H  G  K  A  L      71
122      TTCCGTTCTGGTACCTCTGATATCGTCCTTCCCCATAAAGTTGCACATGGTAAGGCACTG
     L  Y  N  G  Q  K  N  R  G  P  V  A  T  G  V  V  G  V  I  A      91
182     CTCTACAACGGTCAGAAAAATCGTGGTCCAGTTGCGACTGGCGTGGTTGGAGTAATTGCT
     Y  S  M  S  D  G  N  T  L  A  V  L  F  S  V  P  Y  D  Y  N      111
242      TATTCCATGAGCGATGGAAACACCCTGGCCGTTTTGTTCAGCGTTCCCTATGACTATAAC
     W  Y  S  N  W  W  N  V  R  V  Y  K  G  Q  K  R  A  N  Q  R      131
302    TGGTACAGCAACTGGTGGAATGTTAGGGTCTATAAAGGACAAAAACGAGCAAACCAGAGG
     M  Y  E  E  L  Y  Y  H  R  S  P  F  R  G  D  N  G  W  H  S      151
362     ATGTACGAGGAACTCTACTACCATCGGTCTCCATTTCGAGGGGACAATGGCTGGCACTCC
     R  S  L  G  Y  G  L  K  S  R  G  F  M  N  S  S  G  H  A  I      171
422     AGGAGCCTTGGATATGGATTGAAGAGCCGTGGATTCATGAACAGCTCTGGACATGCCATA
     L  E  I  H  V  T  K  A  *                                       179
482     CTGGAAATTCACGTGACCAAAGCTTAAGATCTTGTTGAAAACAAATCAATTGAAATGCTT
                          HindIII
542  CCCCGAGGAAACTGATGTAAAACTAGCTAAAAGACTCTAATTTTACCCTGTAAGACAAAA
602  AACCTAGATCTTCCCATAACATAAAGACAAATAAAATGAAGCACCAAAAAAAAAAAAAAA
662  AAAAAAAAATAGGGATCCAATCAG
                 BamHI
```

Figure 2. Partial cDNA and amino acid sequences. **(A)** Fra B. **(B)** FraE. The first 35 nucleotides of FraB, and the first 32 nucleotides of FraE correspond to the primers ol_fra3b and ol_fra1 (Supplementary Table S1), respectively, and the corresponding amino acids are extracted from the N- terminal protein sequence. GenBank accession for FraB and FraE have been deposited under entry codes MK936900 and MK936901, respectively.

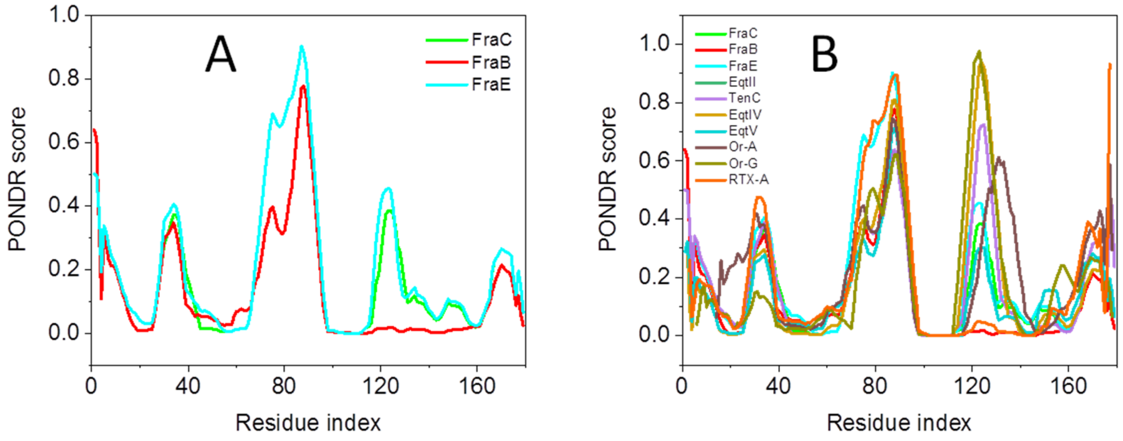

Figure 3. Structure of actinoporins. **(A)** Degree of disorder of residues in FraB, FraC, and FraE and **(B)** in several actinoporins. The disorder score (Y-axis) was computed with PONDR VLTX [1]. Large PONDR scores correspond to highly disordered residues. In several segments, the profile of FraE perfectly overlaps that of FraC given the high similarity between them.

Table 1. Primer sequences used in the amplification of fragaceatoxin sequences.

Name	Sequence	Restriction site
ol_fra1	5'ATATAT<u>CCATGG</u>CTGACGTTGCTGGTGCTGTTATCGACGG	*Nco*I
ol_fra3b	5'ATATAT<u>CCATGG</u>TTGCTGTTGCTGGTGCTATCATCCAAGGTGC	*Nco*I
ol_pTb	5'CTGATT<u>GGATCC</u>CTATTTTTTTTTTTTTTTTTTTTTTT	*Bam*HI
fwd FraE	5'GAGATATAT<u>CCATGG</u>CAGATGTGGCCGGTGCCGTGAT'3	*Nco*I
rev FraE	5'ATACTC<u>AAGCTT</u>TCAGGCTTTGGTCACATGAATTTCCAGGATGG'3	*Nco*I
fwd dest vector	5'TGA<u>AAGCTT</u>GAGTATTCTATAGTGTCACC'3	*Nco*I
rev dest vector	5'<u>CCATGG</u>ATATATCTCCTTCTTAAAG'3	*Hind*III

Table 2. Data collection and refinement statistics. Statistical values given in parenthesis refer to the highest resolution bin.

**Data Collection**	**FraE**
Space Group	P 1 2_1 1
Unit cell	
a, b, c (Å)	55.0, 42.9, 71.9
α, β, γ (°)	90.0, 97.6, 90.0
Resolution (Å)	36.7 – 2.22
Wavelength	1.0000
Observations	85,841 (12,714)
Unique reflections	16,687 (2,412)
$R_{merge.}$	0.16 (0.59)
$R_{p.i.m.}$	0.078 (0.28)
$CC_{1/2}$	0.987 (0.800)
I / *σ* (*I*)	7.7 (2.6)
Multiplicity	5.1 (5.3)
Completeness (%)	99.9 (100)
**Refinement**	
Resolution (Å)	36.7 – 2.22
Rwork / *Rfree* (%)	18.7 / 23.0
No. protein chains	2
No. atoms	

Protein	2784
Solvent	150
B-factor (Å^2)	
Protein	22.8
Water	23.7
Ramachandran	
Preferred (%)	90.7
Allowed (%)	9.3
Outliers (%)	0
RMSD bond (Å)	0.014
RMSD angle (°)	1.83
PDB entry code	6K2G

Table 3. Number of residues classified according to conservation and accessible surface area (ASA).[a, b, c, d.]

Conservation	High ASA	Partial ASA	Low ASA	Total	%
**Fragaceatoxins**					
Identical	27	46	69	142	79
High	3	3	4	10	6
Low	2	2	0	4	2
None	12	9	2	23	13
Total	44	60	75	179	100
**Actinoporins**					
Identical	10	20	35	65	36
High	5	14	22	41	23
Low	3	4	8	15	8
None	26	22	10	58	32
Total	44	60	75	179	100

[a] The degree of conservation was calculated with Clustal Omega [2]. [b] ASA values of side-chains were calculated form the crystal structure of FraC pore (4TSY) using GETAREA [3] and refer to the ratio between the ASA of the side-chain and the ASA of the residue in random coil conformation. High (ASA ≥ 50 %), partial (ASA from 21 49 %), and low ASA (ASA ≤ 20 %) correspond to different degrees of side- chain exposure to solvent [4]. [c] The first, second, and third residues in the sequence were assigned a highly accessible value given their disorder disposition in the crystal structure (4TSY). [d] Cells are color-coded according to residue abundance from low (light orange) to high (dark orange).

Table 4. Number of non-interacting residues classified according to conservation and accessible surface area (ASA).[a, b, c, d, e.]

Conservation	High ASA	Partial ASA	Low ASA	Total	%
**Fragaceatoxins**					
Identical	19	28	48	95	75
High	2	3	2	7	6
Low	2	2	0	4	3
None	12	7	2	21	17
Total	35	40	52	127	100
**Actinoporins**					
Identical	6	13	24	43	34
High	4	10	16	30	24
Low	3	4	7	14	11
None	22	13	5	40	31

Total	35	40	52	127	100

[a] The degree of conservation was calculated with Clustal Omega [2]. [b] ASA values of side-chains were calculated form the crystal structure of FraC pore (4TSY) using GETAREA [3] and refer to the ratio between the ASA of the side-chain and the ASA of the residue in random coil conformation. High (ASA ≥ 50 %), partial (ASA from 21 to 49 %), and low ASA (ASA ≤ 20 %) correspond to different degrees of side- chain exposure to solvent [4]. [c] The first, second, and third residues in the sequence were assigned a highly accessible value given their disorder disposition in the crystal structure (4TSY). [d] Cells are color-coded according to residue abundance from low (light orange) to high (dark orange).[e] Non-interacting residues are shown in Figure 6 and were obtained from [5].

Table 5. Number of interacting residues classified according to conservation and accessible surface area (ASA).[a, b, c, d, e.]

Conservation	High ASA	Partial ASA	Low ASA	Total	%
**Fragaceatoxins**					
Identical	7	19	21	47	90
High	1	0	2	3	6
Low	0	0	0	0	0
None	1	1	0	2	4
Total	9	20	23	52	100
**Actinoporins**					
Identical	4	7	11	22	42
High	1	4	5	10	19
Low	0	0	2	2	4
None	4	9	5	18	35
Total	9	20	23	52	100

[a] The degree of conservation was calculated with Clustal Omega [2]. [b] ASA values of side-chains were calculated form the crystal structure of FraC pore (4TSY) using GETAREA [3] and refer to the ratio between the ASA of the side-chain and the ASA of the residue in random coil conformation. High (ASA ≥ 50 %), partial (ASA from 21 to 49 %), and low ASA (ASA ≤ 20 %) correspond to different degrees of side- chain exposure to solvent [4]. [c] The first, second, and third residues in the sequence were assigned a highly accessible value given their disorder disposition in the crystal structure (4TSY). [d] Cells are color-coded according to residue abundance from low (light orange) to high (dark orange).[e]Interacting residues are shown in Figure 6 and were obtained from [5]. These residues correspond to the sum of the lipid-binding and protein-binding residues. Note that residues in positions 56, 79, 166, and 167 have both lipid and protein interacting partners (Figure 6).

Table 6. Number of protein-binding residues classified according to conservation and accessible surface area (ASA).[a, b, c, d, e.]

Conservation	High ASA	Partial ASA	Low ASA	Total	%
**Fragaceatoxins**					
Identical High<br>Low<br>None	1	10	11	22	92
	0	0	1	1	4
	0	0	0	0	0
	0	1	0	1	4
Total	1	11	12	24	100
**Actinoporins**					
	0	2	4	6	25

Identical High	0	2	4	6	25
Low	0	0	2	2	8
None	1	7	2	10	42
Total	1	11	12	24	100

[a] The degree of conservation was calculated with Clustal Omega [2]. [b] ASA values of side-chains were calculated form the crystal structure of FraC pore (4TSY) using GETAREA [3] and refer to the ratio between the ASA of the side-chain and the ASA of the residue in random coil conformation. High (ASA ≥ 50 %), partial (ASA from 21 to 49 %), and low ASA (ASA ≤ 20 %) correspond to different degrees of side- chain exposure to solvent [4]. [c] The first, second, and third residues in the sequence were assigned a highly accessible value given their disorder disposition in the crystal structure (4TSY). [d] Cells are color-coded according to residue abundance from low (light orange) to high (dark orange). [e] Protein-binding residues are shown in Figure 6 and were obtained from [5].

Table 7. Number of lipid-binding residues classified according to conservation and accessible surface area (ASA).[a, b, c, d, e.]

Conservation	High ASA	Partial ASA	Low ASA	Total	%
**Fragaceatoxins**					
Identical	7	10	12	29	91
High	1	0	1	2	6
Low	0	0	0	0	0
None	1	0	0	1	3
Total	9	10	13	32	100
**Actinoporins**					
Identical	4	6	8	18	56
High	1	2	1	4	13
Low	0	0	1	1	3
None	4	2	3	9	28
Total	9	10	13	32	100

[a] The degree of conservation was calculated with Clustal Omega [2].[b] ASA values of side-chains were calculated form the crystal structure of FraC pore (4TSY) using GETAREA [3] and refer to the ratio between the ASA of the side-chain and the ASA of the residue in random coil conformation. High (ASA ≥ 50 %), partial (ASA from 21 to 49 %), and low ASA (ASA ≤ 20 %) correspond to different degrees of side- chain exposure to solvent [4].[c] The first, second, and third residues in the sequence were assigned a highly accessible value given their disorder disposition in the crystal structure (4TSY). [d] Cells are color-coded according to residue abundance from low (light orange) to high (dark orange).[e] Lipid-binding residues are shown in Figure 6 and were obtained from [5].

References

1. Uversky, V.N.; Dunker, A.K. Understanding protein non-folding. *Biochim. Biophys. Acta* **2010**, *1804*, 1231–1264, doi:10.1016/j.bbapap.2010.01.017.
2. Sievers, F.; Wilm, A.; Dineen, D.; Gibson, T.J.; Karplus, K.; Li, W.Z.; Lopez, R.; McWilliam, H.; Remmert, M.; Soding, J., et al. Fast, scalable generation of high-quality protein multiple sequence alignments using Clustal Omega. *Mol. Syst. Biol.* **2011**, *7*, doi:10.1038/msb.2011.75.
3. Fraczkiewicz, R.; Braun, W. Exact and efficient analytical calculation of the accessible surface areas and their gradients for macromolecules. *J. Comput. Chem.* **1998**, *19*, 319–333, doi:10.1002/(Sici)1096-987x(199802)19:3<319::Aid-Jcc6>3.3.Co;2-3.
4. Jouiaei, M.; Sunagar, K.; Gross, A.F.; Scheib, H.; Alewood, P.F.; Moran, Y.; Fry, B.G. Evolution of an ancient venom: recognition of a novel family of cnidarian toxins and the common evolutionary origin of sodium and potassium neurotoxins in sea anemone. *Mol. Biol. Evol.* **2015**, *32*, 1598–1610, doi:10.1093/molbev/msv050.
5. Tanaka, K.; Caaveiro, J.M.M.; Morante, K.; Gonzalez-Manas, J.M.; Tsumoto, K. Structural basis for self-assembly

of a cytolytic pore lined by protein and lipid. *Nat. Commun.* **2015**, *6*, doi:10.1038/ncomms7337.
